# Physical Exercise May Increase Plasma Concentration of High-Density Lipoprotein-Cholesterol in Patients With Alzheimer’s Disease

**DOI:** 10.3389/fnins.2020.00532

**Published:** 2020-05-27

**Authors:** Camilla Steen Jensen, Christian Sandøe Musaeus, Ruth Frikke-Schmidt, Birgitte Bo Andersen, Nina Beyer, Hanne Gottrup, Peter Høgh, Karsten Vestergaard, Lene Wermuth, Kristian Steen Frederiksen, Gunhild Waldemar, Steen Hasselbalch, Anja Hviid Simonsen

**Affiliations:** ^1^Danish Dementia Research Centre, Copenhagen University Hospital Rigshospitalet, Copenhagen, Denmark; ^2^Department of Clinical Biochemistry, Copenhagen University Hospital Rigshospitalet, Copenhagen, Denmark; ^3^Department of Clinical Medicine, University of Copenhagen, Copenhagen, Denmark; ^4^Department of Physical and Occupational Therapy, Bispebjerg and Frederiksberg Hospital, University of Copenhagen, Copenhagen, Denmark; ^5^Dementia Clinic, Department of Neurology, Aarhus University Hospital, Aarhus, Denmark; ^6^Regional Dementia Research Centre, Department of Neurology, Zealand University Hospital, Roskilde, Denmark; ^7^Dementia Clinic, Aalborg University Hospital, Aalborg, Denmark; ^8^Dementia Clinic, Department of Neurology, Odense University Hospital, Odense, Denmark

**Keywords:** Alzheimer’s disease, exercise, lipid profile, cholesterol, fitness, HDL-C

## Abstract

Lifestyle factors have been shown to increase the risk of developing Alzheimer’s disease (AD) later in life. Specifically, an unfavorable cholesterol profile, and insulin resistance are associated with increased risk of developing AD. One way to non-pharmacologically affect the levels of plasma lipids is by exercise, which has been shown to be beneficial in cognitively healthy individuals. In this randomized controlled trial y, we therefore aimed to clarify the effect of physical exercise on the lipid profile, insulin and glucose in patients with AD. In addition, we investigated the effect of apolipoproteinE genotype on total cholesterol, high-density lipoprotein-cholesterol (HDL-C), low-density lipoprotein–cholesterol (LDL-C), and triglycerides (TG) in plasma from patients with AD. Plasma samples from 172 patients who underwent 16 weeks of moderate-to-high intensity exercise (*n* = 90) or treatment as usual (*n* = 82) were analyzed change from baseline for the levels of total cholesterol, LDL-C, HDL-C, TG, glucose, and insulin. In addition, we analyzed those from the exercise group who adhered to the protocol with an attendance of 2/3 or more of the exercise session and who followed the protocol of an intensity of 70% of the maximum heart rate. We found a significant increase in plasma HDL-C levels between the “high exercise sub-group” compared to control group. After intervention HDL-C was increased by 4.3% in the high-exercise group, and decreased by 0.7% in the control group, after adjustment for statin use. In conclusion, short term physical activity may be beneficial on the cholesterol profile in patients with AD.

## Introduction

Lifestyle risk factors in midlife are associated with risk of developing Alzheimer’s disease (AD) later in life (Kivipelto et al., 2001; Pope et al., 2003; Biessels et al., 2006). In particular, an adverse lipid profile, and insulin resistance have been associated with increased risk of developing AD. The exact role of lipids and lipoproteins for development of AD or AD pathology is, however, unknown (Anstey et al., 2017). Previous studies are conflicting and show both increases and decreases risk associated with high levels of total cholesterol. Studies show that increased TC in mid-life increases risk of AD. However, in late-life, TC was not associated with cognitive or dementia outcomes in any analyses or in any of the large individual studies that were not compatible for pooling (Anstey et al., 2017). Further, high-density lipoprotein-cholesterol (HDL-C) levels in aging individuals have been associated with better cognition. Taken together, modifications of the lipid profile e.g., decreasing total cholesterol and low-density lipoprotein–cholesterol (LDL-C) and increasing HDL-C concentrations might benefit patients with AD.

The importance of lipids and lipoproteins in AD is further underlined by the involvement of a key lipid-transport protein, apolipoproteinE (apoE) in risk of late-onset AD (Di Paolo and Kim, 2011; Liao et al., 2017). The *APOE* gene is polymorphic and three common alleles (ε2, ε3, and ε4) code for three major protein isoforms (Mahley and Rall, 2000). Individuals with ε4/ ε4 have an 8–10-fold higher risk of developing AD as compared with ε3/ ε3 in general population samples (Rasmussen et al., 2015), and the ε4-allele may also be associated with more aggressive subtypes of AD (Høgh et al., 2001). A stepwise decrease in plasma levels of apoE is observed from ε2 to ε3 to ε4, and recently three prospective studies reported that low plasma apoE levels were associated with high AD risk, independent of the ε2/ε3/ε4 polymorphism (Rasmussen et al., 2015; Wolters et al., 2016). Further, apoE is pivotal in peripheral lipid metabolism by serving as a ligand for members of the LDL receptor family mediating hepatic uptake of atherogenic triglyceride-rich lipoproteins (Rasmussen et al., 2020). It is well-established that the APOE polymorphism is associated with all major lipid and lipoprotein classes with a more atherogenic lipid profile from ε2 to ε3 to ε4. The ability to modify plasma levels of lipids and lipoproteins may therefore depend on the individual *APOE* genotype.

Among other metabolic changes that may be implicated in AD, several studies have found an association between cognitive decline or dementia, and diabetes (Yaffe et al., 2006). Several mechanisms have been proposed to explain the association between cognition and glucose control, yet there is still no consensus regarding the biological pathways involved (Yaffe et al., 2006; Ravona-Springer et al., 2010). Even though the brain is not reliant on insulin for glucose uptake (Gray et al., 2014), insulin appears in the cerebrospinal fluid (CSF), and one hypothesis is that insulin acts as a signaling peptide in the brain (Gray et al., 2014).

Due to adverse side effects of pharmacological treatments for metabolic syndromes, a non-pharmacological approach is worth exploring (Wang and Xu, 2017). Here, physical exercise has been shown to have beneficial effects on HDL-C and total cholesterol levels in plasma in cognitively healthy individuals (Haskell, 1984; Casella-Filho et al., 2011). Furthermore, exercise has been shown to increase insulin sensitivity and uptake of glucose in muscle in both young and older adults (Peirce, 1999). Yet, the effect of exercise on the metabolism in AD is largely unknown (Jensen et al., 2015).

In this study, we aimed to explore the effect of physical exercise on the lipid profile, levels of insulin and glucose, and the effect of *APOE* genotype on cholesterol metabolism in plasma from patients with AD. The patients included in this study participated in a 16-week exercise program physical exercise. We hypothesized that the physical exercise would have a beneficial effect on the plasma lipid profile of the AD patients, by lowering plasma levels of TGs and increasing HDL-C concentration. In an exploratory manner, we also investigated the changes in plasma insulin and glucose.

## Materials and Methods

### Study Population

**FIGURE 1:**
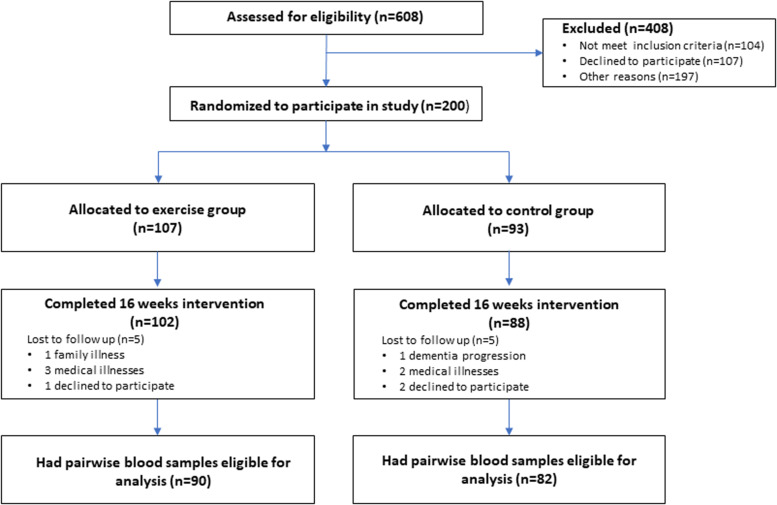
Flowchart of screening and enrolment process. A graphical representation of the screening process of patients seen in the eight participating memory clinics around Denmark. From the screened 608 patients 408 did not meet inclusion criteria o declined to participate. In total 200 patients were randomized to either a control group or an intervention group. One-hundred nighty patients completed the 16 weeks of intervention. The 10 lost to follow up where either due to medical illnesses or declining to participate in the follow up assessment. From the 190 completing patients, matched blood samples (baseline and follow up) where available from 172 patients in total.

This study is part of the previously published ADEX trial study (Hoffmann et al., 2016). The enrollment of participating patients is illustrated in Figure 1. In short, the study population were recruited from eight memory clinics around Denmark and consisted of patients referred for examination of cognitive problems. In total, 198 community-dwelling patients diagnosed with mild AD according to NINCDS-ADRDA criteria (McKhann et al., 1984), with a mini mental state examination (MMSE) >19 and who met the additional inclusion criteria as described in Hoffmann et al. (2013) were included. Patients were randomized to either a control group with treatment as usual or an intervention group. The intervention consisted of a 16 weeks program of three 60-min sessions per week. During the last 12 weeks the participants performed moderate-to-high intensity aerobic physical exercise in groups of four to six persons, supervised by a trained physical therapist. The target exercise intensity was 70% of maximal heart rate (mHR) or higher (Sobol et al., 2016). Patients who participated in more than 2/3 of the exercise sessions and who had a mean intensity of 70% or higher of mHR were named “high exercise group.” For detailed description of the inclusion/exclusion criteria, the exercise intervention used and samples size estimation see Hoffmann et al. (2013). The study is approved by The Danish Regional ethics Committee Capital Region of Denmark H32011128. Baseline characteristics of the study population can be seen in Table 1.

**TABLE 1:** Baseline characteristics of the study cohort.

	Controls (*n* = 82)	Exercise (*n* = 90)	*p*-value^€^	*t*-value	High-exercise sub-group (*n* = 58)	*p*-value^$^	*t*-value
Age, years^#^	71.3 (7.5)	69.8 (7.5)	0.207	1.266	69.9 (7.6)	0.316	1.007
Gender, *n* (%)			0.189			0.153	
Males	51 (62)	47 (52)			29 (50)		
Females	31 (38)	50 (48)			29 (50)		
Characteristics							
Disease duration, years from diagnosis^#^	1.3 (1.1)	1.0 (1.0)	0.085	1.735	0.9 (0.8)	0.061	1.886
MMSE^#^	24.2 (3.8)	23.9 (3.5)	0.579	0.556	24.2 (3.3)	0.994	−0.008
Education, years^#^	11.7 (2.7)	11.9 (2.8)	0.746	−0.325	12.2 (2.8)	0.338	−0.962
BMI^#^	24.2 (3.6)	25.0 (3.7)	0.150	−1.446	24.6 (3.8)	0.495	−0.685
Medications, n yes (%)							
Hypertension	34 (41)	40 (44)	0.695		20 (34)	0.407	
Statins	29 (35)	33 (37)	0.694		19 (33)	0.483	
Diabetes	6 (7)	8 (9)	0.743		5 (9)	0.064	
*APOE*ε*4*, n(%)							
Carriers	62 (77)	56 (68)	0.059		39 (71)	0.280	
Non-carriers	20 (23)	34 (32)			19 (29)		

*^#^Given as mean (Standard deviation), ^€^Controls versus exercise, ^$^Controls versus high exercise. From the exercise group, participants who participated more than 2/3 of the session and had a mean intensity of 70% or high, were further analyzed as a “high exercise sub- group.” MMSE: mini mental state examination, BMI: body mass index.*

### Samples

Blood samples were collected before and after the intervention period. Samples were collected according to standard guidelines (Vanderstichele et al., 2011; del Campo et al., 2012; Teunissen et al., 2014), centrifuged at 2000 G immediately after collection, aliquoted, and stored at –80°C.

### Assays

Collected plasma samples from 172 patients, subjected to 16 weeks of moderate-to-high intensity exercise (exercise group, *n* = 90), or treatment as usual (control group, *n* = 82) were analyzed at baseline and after 16 weeks of exercise. Plasma levels of total cholesterol, LDL-C, HDL-C, triglycerides (TG), glucose, and insulin were measured with standard hospital assays on COBAS 8000 equipment (Roche, Basel, Switzerland).

### Apolipoprotein E Genotyping

DNA was isolated from 250 μL of buffy coat from 6 mL EDTA vials with Promega Maxwell DNA purification kits (Promega, WI, United States), according to the manufacturer’s protocol. *APOE* genotyping for the ε2, ε3, and ε4 alleles was performed with a TaqMan qPCR assay as described by Koch et al. (2002).

### Statistics

All statistics were performed in MATLAB (vR2016a) and IBM SPSS Statistics (Version 24). To compare gender, APOE status, and medications (anti-hypertensive, statins, and diabetes medication), we performed chi-square tests between the control and the exercise groups, and between the control and high exercise groups. To test for differences in continuous variables between the control and the exercise group, we performed *t*-tests for the following values: age, years of education, MMSE score, disease duration, and body mass index (BMI). We also used *t*-tests to compare MMSE and BMI between baseline and follow-up for the exercise and high exercise groups. Only patients who had a baseline and a follow up plasma sample available for analysis were included in the analysis in Table 2.

**TABLE 2:** Changes in plasma biomarkers after 16 weeks of intervention.

	Baseline		16 weeks follow up		Mean relative change from baseline, [(16 week follow up – Baseline)/Baseline]*100				
			
	Controls	Exercise	Controls	Exercise	Controls	Exercise	*p*-value	*High exercise*	*p*-value
	(*n* = 82)	(*n* = 90)	(*n* = 82)	(*n* = 90)	(*n* = 82)	(*n* = 90)		(*n* = 58)	
Total cholesterol, mmol/l	5.6 (1.1)	5.4 (1.0)	5.6 (1.2)	5.5 (1.1)	1.5 (13.1)	2.1 (14.5)	0.81	0.6 (12.0)	0.68
LDL-C, mmol/l	3.1 (0.9)	3.0 (0.9)	3.1 (1.0)	3.0 (0.9)	2.2 (21.0)	2.2 (28.2)	0.96	−1.2 (20.8)	0.36
HDL-C, mmol/l	1.8 (0.6)	1.7 (0.5)	1.8 (0.5)	1.7 (0.5)	−0.7 (13.8)	3.0 (13.5)	0.09	4.3 (13.0)	0.03*
Triglyceride, mmol/l	1.2 (0.6)	1.4 (0.6)	1.2 (0.6)	1.4 (0.6)	1.9 (30.2)	4.5 (35.8)	0.64	2.4 (27.4)	0.92
Glucose, mmol/l	6.0 (2.6)	5.7 (2.1)	5.7 (2.2)	5.6 (1.1)	−1.5 (20.1)	2.3 (21.8)	0.25	0.7 (24.1)	0.56
Insulin, pmol/l	115.7 (100.1)	119.4 (118.3)	138.3 (152.6)	115.5 (138.9)	54.2 (131.6)	22.4 (98.0)	0.07	26.2 (111.0)	0.18

Baseline and follow-up values of cholesterol, HDL, LDL, insulin, and glucose in mean (standard deviation). In addition, analysis of between group difference in mean change from baseline exercise versus controls and per-protocol high-exercise versus controls. HDL-C: high-density lipoprotein-cholesterol, LDL-C: Low-density lipoprotein-cholesterol, *indicates significant difference (p-value < 0.05). A positive value means greater positive mean change from baseline in the exercise group.

Before performing any statistical tests, we calculated the change from baseline by dividing the value at follow-up with the value at baseline times a hundred. To compare the lipids (total cholesterol, LDL-C, HDL-C, and TG), we performed an ANCOVA between the control group and the exercise group, and between the control group and high exercise group using statins as a covariate. The same analyses were performed for insulin and glucose but with current use of diabetes medication as a covariate instead of statins. In the current study, we also wanted to investigate the effects of exercise depending on the *APOE* genotype. When looking at the effect of exercise depending on the *APOE* genotype for lipids, we performed an ANOVA for each of the lipids. A statistical test was considered significant if the *p*-value was below 0.05. No corrections for multiple comparisons were performed due to the exploratory nature of the study.

## Results

Baseline characteristics of the control group, exercise group, and high exercise sub-group were comparable, as shown in Table 1. The mean age of the patients in the two group was similar, and the mean MMSE was comparable with 21.1 for the controls and 22.4 for the exercise group, which categorizes the cohort as mild-to- moderate AD (Budd et al., 2011). There was no significantly differences between the groups with regards any of the population characteristics, including MMSE and BMI, and no significant differences in MMSE and BMI between baseline and follow-up in either the exercise or the high exercise group. In addition, there were no significantly differences between the groups with regards to use of dementia medication, statins or anti-diabetics. The *APOE* genotype regarding the ε4 allele is also listed in Table 1. Seventy-two per cent of the subjects were carriers of either one or two *APOE* ε4 alleles of, which is higher than the expected 60% in an AD population (Ward et al., 2012).

As published previously there was a good over all adherence to the trial, with a drop out of only 5%. Adherence to the protocol in the exercise group (termed “High exercise sub-group”) was 65% (Hoffmann et al., 2016). Overall the trial was well tolerated with few adverse events (Hoffmann et al., 2016).

No significant differences were found for the levels of total cholesterol, LDL-C, HDL-C, TG, glucose, and insulin at baseline between the groups, see Table 2. We found a significant increase in plasma HDL-C levels between the “high exercise sub-group” compared to control group. After intervention HDL-C was increased by 4.3% (SD 13.0) in the high-exercise group and decreased by 0.7% (SD 13.8) in the control group *p* = 0.03, after adjustment for statin use. No difference was observed for the other markers, as these remained stable over the 16 weeks of intervention.

**FIGURE 2:**
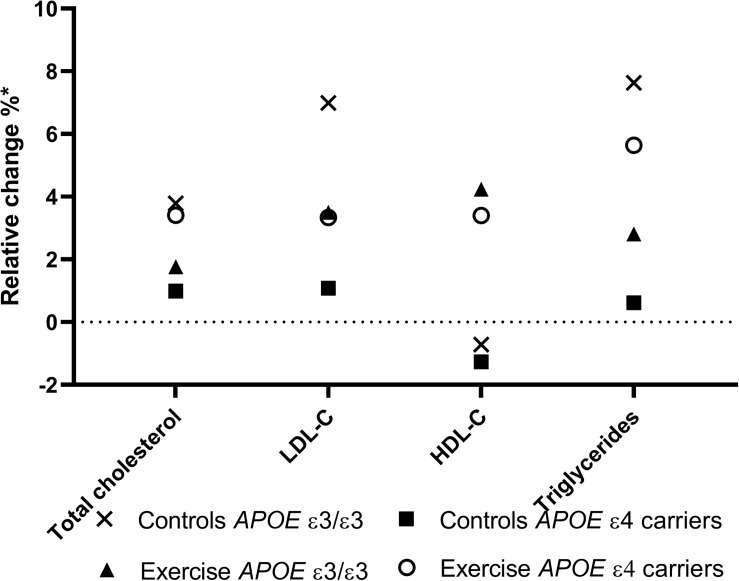
Mean allelic effect of relative change in lipid concentration in plasma. A graphical representation of the effect of exercise on the relative change from baseline in triglycerides, Low-density lipoprotein, cholesterol, and High-density lipoprotein, segregated by *APOE* ε4 carriers and non-carriers (ε3/ε3). The *APOE*ε*2* allele is here excluded. Figure legend: X:control groups *APOE* ε3/ ε3, open circle: Exercise group *APOE* ε4 carriers, +: exercise *APOE* ε3/ ε3, and closed squares: control group *APOE* ε4 carriers.* from baselin.

To elucidate the effect of the *APOE* ε4 genotype on the effect of exercise on the lipid profile in patients with AD, the results were divided and analyzed in groups of non-carriers versus carriers of the ε4 allele. This analysis revealed no significant difference between the control group and the exercise group in either *APOE* ε4 carrier and non-carriers, see Table 3. Due to low sample size (*n* = 13) the ε2 genotype (ε2/ε2, ε2/ε3, and ε2/ε4) was excluded in this analysis. Figure 2 is a graphical representation of the results in Table 3. Here, the mean relative change from baseline is plotted for each group and according to *APOE* genotypes for each of the four lipid outcome measures.

**TABLE 3:** Effects of the intervention in APOE ε4 non-carriers and APOE ε4 carriers.

	Control group		Exercise group		
Mean relative change from baseline	*APOE*ε3/ε3 (*n* = 17)	*APOE* ε4 carriers (*n* = 57)	*APOE* ε3/ε3 (*n* = 32)	*APOE* ε4 carriers (*n* = 54)	*p*-value
Total cholesterol	3.79 (10.63)	0.99 (13.87)	1.76 (17.38)	3.41 (12.09)	0.59
LDL-C	6.99 (22.57)	1.08 (20.67)	3.50 (38.13)	3.34 (19.91)	0.30
HDL-C	−0.72 (11.17)	−1.27 (14.42)	4.24 (16.27)	3.40 (11.01)	0.83
Triglycerides	7.64 (30.00)	0.62 (31.11)	2.81 (47.01)	5.64 (29.01)	0.46

*Group differences in mean (standard deviation) change from baseline in exercise group versus control group depending of APOE ε4 carrier status (0 alleles versus 1 or 2 alleles).*

## Discussion

In the current study, we investigated the effect of 16 weeks of moderate-to-high intensity physical exercise on the plasma levels of lipids, glucose, and insulin in patients with AD. In addition, we investigated the potential effect of *APOE* genotype on the response of exercise on lipids. We found that exercise significantly increased the plasma levels of HDL-C in a sub-group analysis of the subjects who adhered to the protocol and exercised with the target intensity of 70% of mHR or more. Furthermore, though not significant, carriers of *APOE* ε4 allele showed less modulation of the lipid profile after exercise as compared to ε4 non-carriers. We found no differences in glucose and insulin levels between the control and exercise groups.

Individuals with an unfavorable lipid-profile have been shown to have a higher risk of developing AD (Panza et al., 2006), while high HDL-C levels in aging individuals have been associated with better cognition (Crichton et al., 2014; Bates et al., 2017). Among non-pharmacological interventions, exercise has previously been proven beneficial and shown to reduce LDL and increase HDL across all ages in cognitively normal individuals (Kelley et al., 2005). Yet, it is not known whether the effect of exercise has any beneficial effects on AD pathogenesis. Here, we found that exercise significantly increased HDL levels, but no significant effects on the LDL, TG or total cholesterol levels were observed. One reason for this result could be that HDL-C has been shown to be more sensitive to exercise with an increase even present after only a single exercise session (Kodama et al., 2007). Moreover, the increase in HDL-C could be directly linked to how much the AD patients improved in physical fitness, greatest increase in physical fitness would lead to largest effect on HDL-C. The effect on fitness was investigated in a previous study on the same patient cohort, see Sobol et al. (2016). This is also supported by the current study as the largest effect was seen in the high exercise group. The exact role of HDL-C in regard to AD is not fully elucidated. It has been suggested that HDL-C is implicated in amyloid β42 clearance (Zlokovic et al., 2000), exhibits vasoprotective properties through the apolipoproteinI, and modulates inflammation, all of which may be implicated in the AD pathogenesis (Button et al., 2019). This might indicate that an increase in HDL-C levels could be beneficial for patients with AD. Plasma TG levels are also thought to be affected by exercise, but longer intervention periods may be needed for this effect. A study has shown that the beneficial effect on triglyceride levels is proportional with the exercise intensity, and that the intensity of the exercise employed should be high, and lastly no effect was observed on the LDL levels (Wang and Xu, 2017). If this also is the case in patients with AD, we should have seen an effect on the biomarkers investigated in this study when comparing controls versus the high-exercise sub-group. Besides the intervention period possibly being too short to detect changes in TG or LDL-C, the participants did not adhere to a standardized diet, which may affect the plasma concentration of multiple lipids. We did not find any significant differences in BMI over the time course of the study and BMI did not significantly affect our findings of increased HDL, but we did not control for dietary changes, which may have affected our findings. However, we showed that even a short intervention period is able to modulate the HDL-C concentration in plasma, which may be beneficial to patients with AD.

A small decrease in the levels of plasma HDL-C In the control group of 0.7% was observed. Previous studies of longitudinal changes in HDL-C concentrations in the elderly have found conflicting results. Some find that the HDL-C levels are stable, others find that levels either decrease or increase with age (Ferrara et al., 1997). In this study, however, it seems that HDL-C levels do decrease over time in a cohort of elderly patients. A decrease in HDL-C levels might be due to for instance weight loss or a decrease intake of a fatty diet. By analyzing BMI in the control group at baseline and at follow up (data not shown), we did not find a significant change in BMI, reflecting no sign of either weight loss or weight gain. We did not control for dietary intake, and subjects were told to act as normal, here under also regarding food intake.

Studies have found that total cholesterol and LDL increase with age until the age of 65 years for men and 75 years for women, and thereafter the levels start to decline (Ferrara et al., 1997). For HDL-C and TGs, the change is less profound during the lifespan, but there is a tendency to higher levels of HDL-C and TGs in elders, however, longitudinal data is inconsistent (Ferrara et al., 1997). As previously mentioned, studies have found that patients with AD have lower levels of HDL-C compared to age matched cognitively healthy persons. When comparing mean values for HDL-C in this study with results from previous studies, our results are in line with mean findings from populations studies where a mean level of HDL of 1.55 mmol/L compared to our finding with a mean of 1.8 mmol/L in the control group and 1.7 mmol/L in the exercise groups at baseline (Ferrara et al., 1997).

The AD risk factor *APOE* ε4 showed no significant effect on the lipid outcome measurement. However, the graphic representation (Figure 2) indicates that physical exercise for ε4 non-carriers had a tendency toward a positive effect on the lipid profile, with decreasing levels of TG, LDL, and cholesterol, while increasing the HDL levels. On the contrary, carriers of *APOE* ε4 showed less modulation of the lipid profile after exercise when compared to ε4 non-carriers. This is somewhat surprising as the same cohort of *APOE* ε4 carriers benefitted the most from exercise regarding physical outcomes such as walking speed, endurance, and overall fitness (Jensen et al., 2019). Still, it must be stressed that none of these results are statistically significant, and that the number of patients who were ε4/ε4 carriers was low. Since APOE is the major lipid-transporting molecule, it could be speculated that the lack of APOE might have significant effect on the lipid profile (Verghese et al., 2011). The lack of effect on the lipid profile in the *APOE* ε4 carriers could therefore be due to the low plasma levels of APOE. However, larger studies are needed to investigate the effects of exercise depending on *APOE* genotype.

The present study has some limitations. A total of 198 patients were enrolled in the trial, but the number of samples available for analysis was less (*n* = 172). Especially, the subgroup analysis was affected by of the small number of patients in certain subgroups. Furthermore, large variations were seen in the measured markers, which most likely were due to biological variations. Moreover, a large proportion of the subjects was taking statins (control group = 34%, exercise group = 33%), which could influence the obtained results. Therefore, to overcome this issue, we used statin use as a covariate in the statistical analysis. The main issue with the current study may be that exercising for 16 weeks might not have been long enough to induces chances in any of the other markers besides HDL-C, or perhaps the exercise intensity should be higher in order to show changes in metabolism. Other exercise trials with AD patients have been as long as 52 weeks (van Uffelen et al., 2008) whereas increases in HDL-C levels have been observed after only one session of exercise (Segal et al., 2012).

As published previously the trial was well tolerated with few adverse events (Hoffmann et al., 2016). Moderate to high intensity exercise employed in the trial was selected on the basis of similar previous studies in patients with mild cognitive impairment (Baker et al., 2010). In short, the exercise intervention consisted of aerobic exercise on either treadmill, stationary bikes, or cross trainers. Adherence to the exercise protocol was ensured by monitoring and supervision of every training session by experienced trainers, and every participant wore pulse watches on every training session. We were not able to control the activity of the control group or in the exercise group outside the structured interventions. However, both caregiver and patient were instructed to carry on as normal, but no formal control was applied due to lack of resources. If anything, increased physical activity in the control group would have resulted in underestimation of the effect of exercise. Another issue was the impact of social contact by exercising in groups which was not controlled for.

As mentioned above dietary intake of fat can modulate the levels of lipids in plasma. The patients participating in this study did not adhere to a specific diet, and blood samples taken ad baseline and follow up was not taken fasting (Mora, 2016). Long term calorie restriction in normal weight adults has been previously shown to reduce plasma levels of TC, and LDL-C, and to increase levels of HDL-C (Garry et al., 1992). In this study we did not analyze a restriction in diet intake, and analysis of baseline versus follow up in BMI reflected that the patients participating did not lose or gain significant weight in either of the groups, indicating that the increases seen here in HDL-C may solely be due to increased activity levels, here aerobic physical exercise. In addition, have previous studies found that weight loss due to suboptimal energy intake is common in AD (Poehlman and Dvorak, 2000).

In conclusion, in the current study, we found that patients with AD showed significantly increased levels of HDL after only 16 weeks of physical exercise. Compared to other studies with healthy individuals, we did not find any changes in TG or LDL-C levels, which could be due to a too short intervention time. Furthermore, a possible difference in the lipid response to exercise based on the APOE genotype was observed, but due to the low sample size, no definite conclusions can be made. Additionally, exercise showed no effect on glucose and insulin levels in the AD patients. Future studies should investigate the long-term effects of exercise on the lipid profile in patients with AD to get a better understanding of the beneficial effects of exercise.

## Data Availability Statement

The datasets generated for this study will not be made publicly available Danish legislation prohibits this.

## Ethics Statement

The studies involving human participants were reviewed and approved by De videnskabsetiske Komitter i Region Hovedstaden. The patients/participants provided their written informed consent to participate in this study.

## Author Contributions

CJ, GW, SH, and AS contributed to the study design. RF-S contributed to assay performance. BA, NB, HG, PH, KV, LW, KF, and SH contributed to the collection of patient data and samples. CJ and CM contributed to data analysis and interpretation. All authors contributed to manuscript preparation.

## Conflict of Interest

The authors declare that the research was conducted in the absence of any commercial or financial relationships that could be construed as a potential conflict of interest.
